# Phylogenetic analysis and molecular structure of NS1 proteins of porcine parvovirus 5 isolates from Mexico

**DOI:** 10.1007/s00705-024-06182-5

**Published:** 2025-01-24

**Authors:** Alejandro Vargas-Ruiz, Diana Michele Araiza-Hernández, Francisco Rodolfo González-Díaz, Ernesto Marín-Flamand, José Ivan Sánchez Betancourt, Ana Elvia Sánchez-Mendoza, Lucia Angélica García-Camacho

**Affiliations:** 1 Facultad de Estudios Superiores Cuautitlán, Departamento de Ciencias Biológicas, Universidad Nacional Autónoma de México (UNAM), Carretera Cuautitlán–Teoloyucan Km 2.5, Cuautitlán Izcalli, 54714 Estado de México, México; 2 Facultad de Estudios Superiores Cuautitlán, Unidad de Investigación Multidisciplinaria, Universidad Nacional Autónoma de México (UNAM), Estado de México, México; 3 Grupo de Investigación del Departamento de Medicina y Zootecnia de Cerdos, Facultad de Medicina y Zootecnia, Universidad Nacional Autónoma de México, Mexico City, Mexico

## Abstract

Porcine parvovirus 5 (PPV5) is an unclassified member of the family *Parvoviridae* with no reported pathogenicity, although it is associated with multisystemic, reproductive, and respiratory diseases. Its open reading frame 1 (ORF1) encodes non-structural protein 1 (NS1), which is predicted to have helicase activity that is essential for viral replication. This protein contains a C-motif with an invariant asparagine residue that forms the core of the enzyme's active site, in conjunction with the Walker A and B motifs. The aim of this study was the phylogenetic and molecular characterization of the NS1 of PPV5 through nested PCR and sequencing of three Mexican PPV5-positive samples. Subsequently, a phylogenetic tree, identity matrices of nucleotide and amino acid sequences, and a three-dimensional model of NS1 were constructed. The amplified sequences, which represented 96.9% of the PPV5 ORF1, occupied the same branch in the phylogenetic tree and exhibited the most nucleotide sequence similarity to the corresponding region of PPV4 and the most amino acid sequence similarity to the NS1 proteins of PPV4 and PPV6. A three-dimensional model of NS1 displayed a C-motif characteristic of superfamily 3 (SF3) helicases. The phylogenetic proximity of PPV5 to PPV4 and PPV6 suggests that it may belong to the genus *Copiparvovirus*. Further studies on helicases from viruses infecting domestic animals may be useful in developing antiviral drugs for both human and veterinary medicine.

## Introduction

Advanced genome sequencing using porcine tissues has led to the identification of several emerging porcine parvoviruses (PPVs) with distinct geographical distributions [1], belonging to the subfamily *Parvovirinae* of the family *Parvoviridae.* These viruses belong to the species *Protoparvovirus ungulate1* (PPV1), *Tetraparvovirus ungulate2* (PPV3), *Tetraparvovirus ungulate3* (PPV2), *Copiparvovirus ungulate2* (PPV4), and *Copiparvovirus ungulate4* (PPV6) [2]. Recently, PPV7 (species *Chaphamaparvovirus ungulate1*) and porcine parvovirus 8 (PPV8), proposed to belong to the genus *Protoparvovirus*, were identified in rectal swab samples in the USA [3] and lung samples in China [4], respectively. Notably, PPV5 remains unclassified, despite being described previously as a member of the genus *Copiparvovirus* [5–7].

PPV5 has been predominantly detected in cases of reproductive failure (RF) as well as in multisystemic and respiratory diseases [8–10]. PPV5, PPV2, and PPV3 are frequently associated with RF outbreaks [8]. Coinfection with PPV5 and porcine reproductive and respiratory syndrome virus (PRRSV) is also frequent in RF cases [11] and significantly higher in pigs with respiratory diseases [9]. Furthermore, PPV5 is commonly found in cases of porcine circovirus-associated disease (PCVAD) in coinfection with other PPVs [9, 12]. In Mexico, a significant relationship between PPV5 and postweaning multisystemic wasting syndrome (PMWS) as well as RF has been observed. In this study, we found the prevalence of PPV2-PPV6 and the rate of coinfection with other viruses to be markedly higher than those reported in other countries [12].

Although it is likely that they originated from a common ancestor, PPVs exhibit a high degree of genetic variability, which accounts for their pathogenic potential [13]. The PPV5 genome is approximately 5805 nucleotides (nt) in length and has two open reading frames (ORFs) that encode the main proteins – nonstructural protein 1 (NS1 [ORF1]) and capsid protein (VP1 [ORF2]) – with lengths of 601 and 991 amino acids (aa), respectively [7]. ORF1 extends from nt 862 to 2667, encoding non-structural (NS) proteins with replicase activity [7]. The larger NS protein, known as NS1, is a multidomain protein that has been shown for some parvoviruses to have helicase, ATPase, endonuclease, and sequence-specific DNA binding activity [14–16]. This multifunctional protein regulates numerous processes that are essential for viral replication, including viral DNA amplification and regulation of gene expression [17]. In closely related viruses such as PPV1, NS1 has been shown to induce apoptosis through the intrinsic pathway [17–19], promoting cell cycle arrest in the G1 and G2 phases, which is prevented by caspase 9 inhibitors [18]. NS1-induced apoptosis can lead to placental lesions, resulting in RF [18] and the production of interleukin 6 (IL6) through activation of NF-κB in a dose-dependent manner [19]. In the case of mink enteritis virus (MEV), the binding site replication origin (aa 1–337), the helicase domain (aa 338–556), and the transactivation domains (aa 557–668) have been shown to induce apoptosis [20].

Helicases are classified into six superfamilies (SFs), with SF1 and SF2 being the most common, whereas hexameric helicases such as SF3 have been documented less frequently. A detailed characterization of the NS1 of adeno-associated virus (AAV), a member of the family *Parvoviridae*, has shown it to be SF3 helicase. In that report, four conserved domains, identified as "N-terminal", "nuclease", "helicase", and "transactive", were identified. The helicase domain also contained a Walker A motif, Walker B motif, and B´-motif, as well as a C-motif, which is found only in SF3 proteins, suggesting that the NS1 helicase may belong to this superfamily [16, 21]. Point mutations in the ATP-binding site of the helicase of canine parvovirus (CPV) have been shown to affect viral replication [22]. In this study, we determined the NS1 sequences of PPV5 isolates from Mexico and performed phylogenetic analysis and protein structure modeling to compare PPV5 to other *Parvoviridae* members and provide information that can contribute to its classification.

## Materials and methods

### Sample selection

Three PPV5-positive samples – two lymph nodes from piglets with post-weaning multisystemic wasting syndrome and one blood sample from a gilt – were used to amplify the complete ORF1 region by nested PCR for sequence determination.

### Primer design

To obtain the complete ORF1 sequence, a set of primers was designed to amplify three overlapping fragments of approximately 600 nt each, using 14 complete ORF1 sequences from different countries, available in the GenBank database (www.ncbi.nlm.nih.gov/genbank). For each fragment, the Primer 3 input program (v3.0.0, Institute for Biomedical Research, Boston, MA) [23] and the BioEdit program (v7.2.5, Ibis Bioscience, Carlsbad, CA) [24] were used for designing and editing primers. The primer sets are listed in Table 1.

**Table 1:** Primers designed based on sequence JX896322 to amplify overlapping ORF1 fragments of PPV5

Fragment	Primers	Position	5´-3´ sequence	Annealing temperature	Amplicon
1	Forward	542	TTTGTCATTTGCGTTTTGGA	58°	740 bp
	Reverse	1281	CATGGGAGCAGATAATTCTTCA		
	Fw nested	572	AGCAGAACTCCGTCGTTTTC		611 bp
	Rv nested	1182	TCCAATTGTAGTCCATGCATAA		
2	Forward	1090	GCATGGAAAAGCACAGATGA	58°	726 bp
	Reverse	1815	TTTCCCTTCTTCCCACCAC		
	Fw nested	1148	TGAAAGAATGTCTTTATGCATGG		620 bp
	Rv nested	1767	AGGAAAATTTGGATTGTTCCAGT		
3	Forward	1677	TGCCACAACAGGAAAAACAA	60°	721 bp
	Reverse	2397	TGCAAAATTCTTACCTGGAACA		
	Fw nested	1707	GGCAATCTGCCATAGCTCA		650 bp
	Rv nested	2356	AATTCTCATAAGCCGCAGGA		

### Nested PCR

Genomic DNA was extracted from each sample, using a QIAamp DNA FFPE Tissue Kit (QIAGEN, Germany) according to the manufacturer’s instructions. The DNA was eluted in 200 μL of molecular-grade water, and its concentration was determined by spectrophotometry using a NanoDrop Lite instrument (Thermo Fisher Scientific Inc., Waltham, Massachusetts, USA). The DNA was stored in 100 ng/μL aliquots at -20°C until use. The lowest limit of detection was determined by serial dilutions (1:2) of the aliquots. Nested PCR reactions in a total volume of 50 µL were performed in a MasterCycler Gradient Thermocycler (Eppendorf, Hamburg, Germany) using a 1X master mix (MyTaq, Bioline, London, UK) containing 2.5 U of Taq DNA polymerase, 1X PCR buffer, 1.5 mM magnesium chloride, 0.2 mM each deoxynucleotide (dNTP), 10 μM each primer, and 50 ng of DNA template (reaction 1) or 5 µL of the first reaction product for nested amplification. The conditions were as follows: initial denaturation at 95°C for 2 min, 40 cycles of denaturation at 94°C for 35 seconds, annealing at the appropriate temperature for each set of primers (Table 1) for 35 seconds, and elongation at 72°C for 1 min, and finally, a 7-minute final extension at 72°C. The PCR products were then separated by electrophoresis in a 1.5% agarose gel stained with ethidium bromide and visualized using a UV-light transilluminator (Apollo Instrumentation, Claremont, California, USA).

### Sequencing

Each amplified product was purified from an agarose gel, using a MinElute Gel Extraction Kit (QIAGEN, Germany), following the manufacturer's instructions. The products were sequenced using a Big Dye Terminator V3.1 Kit (Applied Biosystems, USA) in a 3500 Genetic Analyzer (Applied Biosystems, USA) at the Institute of Cellular Physiology of the National Autonomous University of Mexico.

### Sequence analysis

The sequences were edited and aligned using BioEdit software (v7.2.5, Ibis Bioscience, Carlsbad, CA) [24] to assemble identity matrices of nucleotide and predicted amino acid sequences. Subsequently, a phylogenetic tree was constructed based on the predicted amino acid sequences of classified and unclassified PPVs (Table 2). The MEGA7 program [25] was used to construct a phylogenetic tree using the maximum-parsimony algorithm. The statistical confidence of the topology was assessed by bootstrap analysis, with 1000 repetitions. Bootstrap values over 65 (650) were considered significant. The sequences for this study were deposited in the GenBank database. The conserved motifs of the helicase domain of the Mexican PPV5 amino acid sequences were aligned with those of other *Parvoviridae* members, including AAV, PPV1, and canine parvovirus (CPV; species *Protoparvovirus carnivoran1*) as well as other reference sequences for SF3 helicases [26].

**Table 2:** Reference sequences used for primer design and phylogenetic analysis

Accession number	Virus	Country
U44978	PPV1	Canada
EU200677	PPV2	China
GU938300	PPV3	China
GQ387499	PPV4	United States
KX384823	PPV6	Poland
MG543471	PPV7	China
JX896322	*PPV5	United States
OP021638	*PPV8	China

* Not yet classified by the ICTV

### Protein modeling

Modeling of the NS1 protein was performed using the Swiss-Model program (https://swissmodel.expasy.org/), with 7jsh.1.A as a template. The model was edited and visualized using PyMOL.

## Results

### Polymerase chain reaction

Using nested PCR, specific products of 611 bp (fragment 1), 620 bp (fragment 2), and 690 bp (fragment 3) were amplified. Sequence editing and assembly of fragments from each positive control revealed a linear sequence of 1750 nt, corresponding to 96.9% of the NS1 sequence of PPV5.

### Sequence analysis

**Fig. 1:**
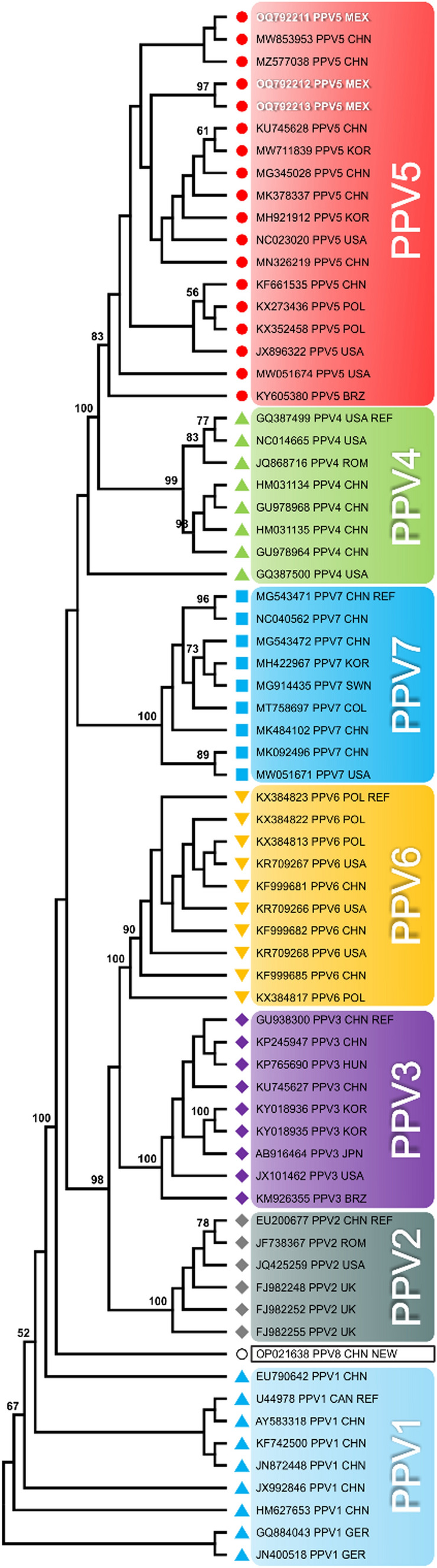
Phylogenetic tree based on NS1 amino acid sequences, showing that the members of porcine parvovirus species were distributed in eight clades: *Protoparvovirus ungulate1* (PPV1), *Tetraparvovirus ungulate3* (PPV2), *Tetraparvovirus ungulate2* (PPV3),  C*opiparvovirus ungulate2* (PPV4),  C*opiparvovirus ungulate4* (PPV6), *Chaphamaparvovirus ungulate1* (PPV7), **○** porcine parvovirus 8 (PPV8), and  unclassified porcine parvovirus 5 (PPV5). The sequences are identified by their accession number and country of origin. The Mexican sequences from this study are highlighted (accession numbers OQ792211, OQ792212, and OQ792213). The tree was constructed using the maximum-parsimony algorithm in MEGA ver. 10.2.4 [25]

A phylogenetic tree based on deduced amino acid sequences of NS1 of porcine parvoviruses (Fig. 1) revealed eight distinct clades, each corresponding to a different PPV

species. These clades were supported by bootstrap values ranging from 80% to 100%. The PPV5 and PPV4 clades originated from the same branch with a bootstrap value of 100. Two of the Mexican PPV5 sequences (OQ792212 and OQ792213) clustered together in an independent branch with bootstrap value of 97, while the third Mexican sequence (OQ792211) grouped with the Chinese sequences MW853953 and MZ577038, forming a distinct branch.

**Fig. 2:**
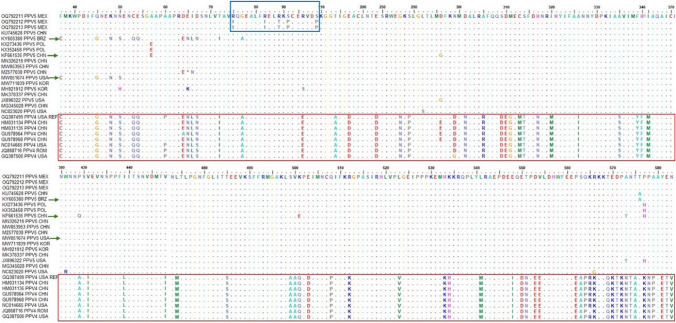
Alignment of NS1 amino acid sequences of PPV5 and PPV4 isolates. Differences between PPV4 and PPV5 sequences are indicated by red boxes, differences among Mexican PPV5 sequences are indicated by a blue box, and differences between the Mexican isolates and other PPV5 isolates are indicated by green arrows. (Graphic view, BioEdit 7.2.5) [24]

The nucleotide sequence identity matrices for the NS1 genes of the Mexican isolates showed that they were 98.6% to 99.6% identical to each other, 96.9% to 99.5% identical to PPV5 sequences from other countries, and 77.6% to 77.9% identical to PPV4 sequences. The aa sequence identity matrices indicated that the Mexican sequences OQ792212 and OQ792213 were 100% identical to each other, 98.4% identical to the Mexican sequence OQ792211, and 96.7% to 100% identical to PPV5 sequences from other countries. Particularly, the Mexican sequence OQ792211 exhibited 100% aa sequence identity to the Chinese sequence MW853953 and 99.6% to the Chinese sequence MZ577038. Conversely, the sequences KY605380 (Brazil) and KF661535 (China) showed the least similarity to the Mexican sequences (96.9% identity), followed by MW051674 (USA) (98.2% identity). In comparison to PPVs of other species, the aa sequences of the Mexican PPV5 isolates showed the most similarity to those of PPV4 (86.2% to 87.3% identity), followed by PPV6 (54.9% to 55.4% identity), and showed the least similarity to those of PPV7 (0.249), PPV2 (0.247), PPV3 (0.258), and PPV1 (0.288). An alignment of the NS1 aa sequences of the Mexican PPV5 isolates and those of PPV5 and PPV4 sequences from other countries is shown in Fig. 2.

### Molecular characteristics of NS1

**Fig. 3:**
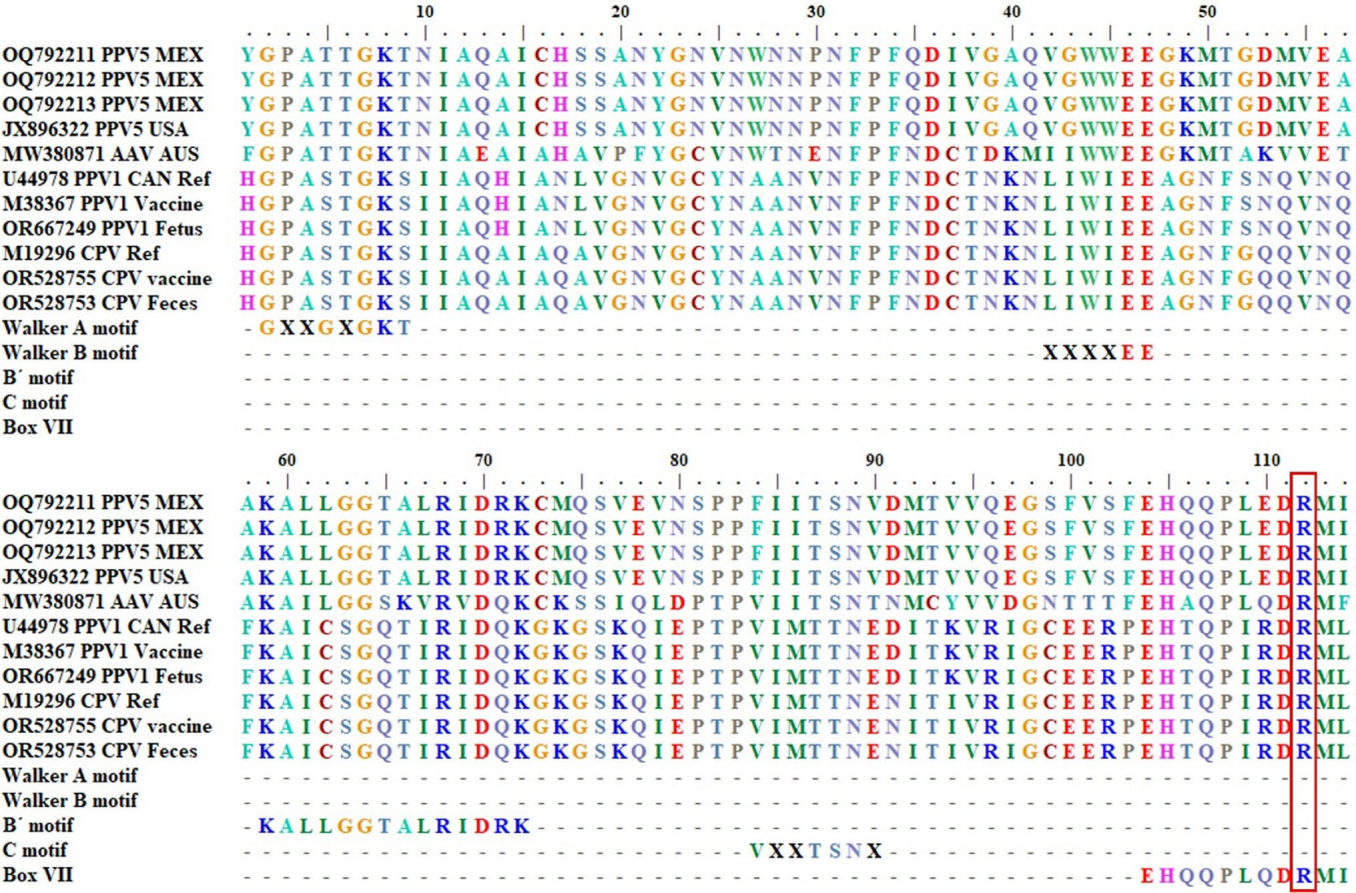
Amino acid sequence alignment of NS1 proteins of *Parvoviridae* family member, showing conserved motifs of the helicase domain of superfamily 3 (SF3). PPV5 (including the Mexican PPV5 sequences), adeno-associated virus (AAV), PPV1 (vaccine and field strains), and canine parvovirus (CPV, vaccine and field strains) are shown. The positions of the motifs in the different viruses are as follows: Walker A (PPV5 355, AAV 335, PPV1 398, and CPV 400), Walker B (PPV5 396, AAV 375, PPV1 438, and CPV 440), B´ motif (PPV5 412, AAV 392, PPV1 455, and CPV 457), C-motif (PPV5 437, AAV 417, PPV1 480, and CPV 482), box VII (PPV5 457, AAV 437, PPV1 500, and CPV 502), and arginine finger (PPV5 465, AAV 445, PPV1 508, and CPV 510). "Ref" indicates an ICTV reference sequence. The red rectangle indicates the arginine finger position. "X" indicates that any amino acid can occupy the position (Graphic view, BioEdit 7.2.5) [24]

The NS1 proteins of the Mexican PPV5 isolates contained the characteristic conserved motifs of helicases, including the C-motif specific for SF3 helicases. The Mexican PPV5 sequences obtained in this study, along with other *Parvoviridae* members, contained conserved motifs from the helicase domain (Walker A, Walker B, B´ motif, C-motif, and box VII with an arginine finger), as shown in Figure 3.

**Fig. 4:**
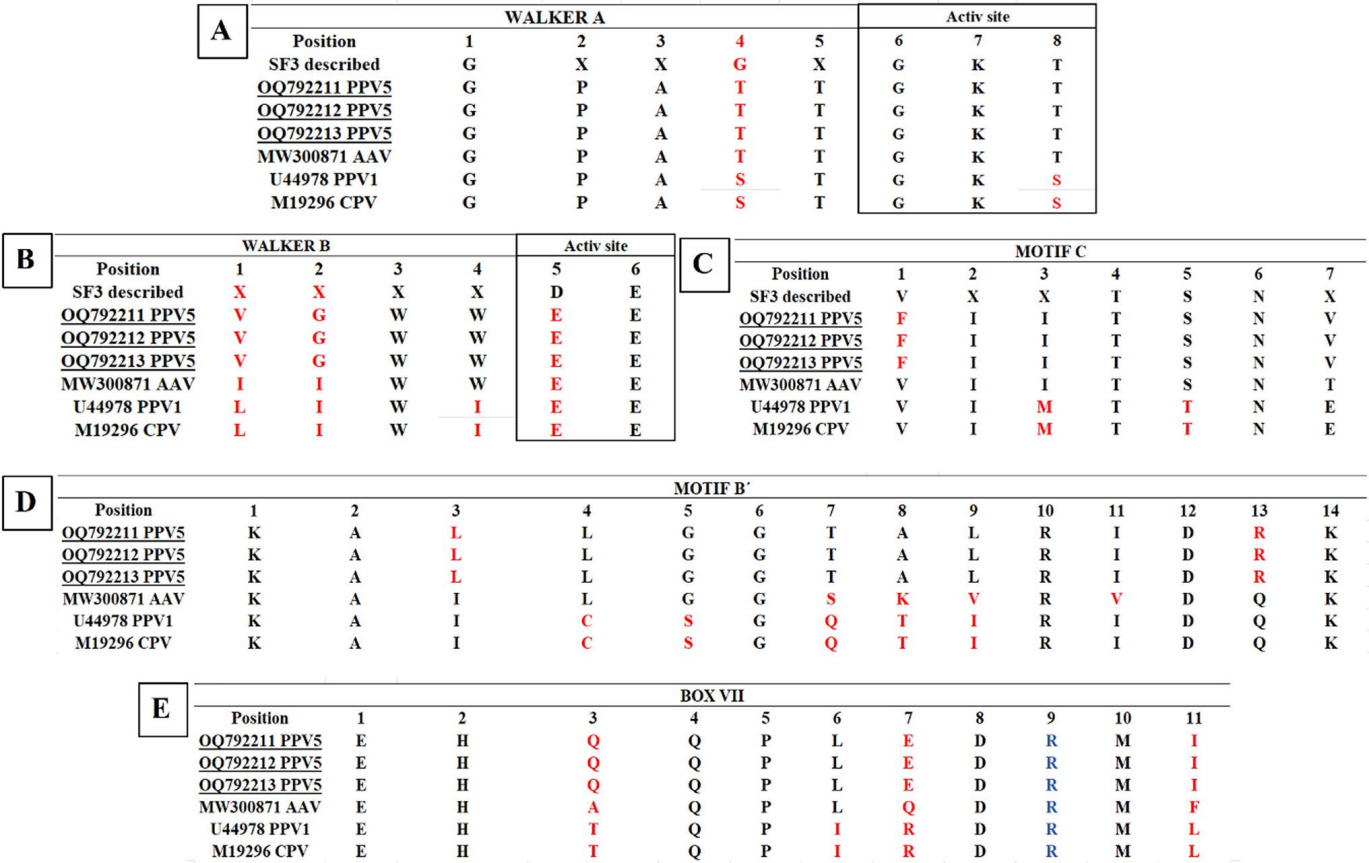
Comparison of conserved motifs of superfamily 3 (SF3) PPV5 (OQ792211, OQ792212, OQ792213) and ICTV reference sequences of AAV, PPV1, and CPV. (A) Walker A motif. (B) Walker B motif. (C) C-motif. (D) B´ motif. (E) box VII. Red letters indicate differences, and blue letters indicate the arginine fingers. X indicates that any amino acid can occupy the position

Although all of the sequences were similar, the current PPV5 and AAV sequences differed in their Walker A SF3 motifs at position 4, where glycine was replaced by threonine (G→T). PPV1 and CPV showed a G→S substitution at that position, as well as a T→S substitution at position 8 within the active site (Fig. 3A). Overall, the Walker B motif exhibited more diversity among the sequences at positions 1, 2, and 5 when compared to the reported SF3 sequence. Furthermore, a change at position 5 (D→E), which is located within the active site, was observed in each sequence (Fig. 4B).

In the C-motif, the Mexican PPV5 sequences exhibited changes at position 1 (V→F), while the PPV1 and CPV sequences differed from the PPV5 sequences at positions 3, 5, and 7 (I→M, S→T, and V→E, respectively). The Mexican PPV5 sequences contained E (glutamic acid) at position 7 of box VII instead of R (arginine), which is present in the PPV1 and CPV sequences. The arginine finger is found in all sequences.

**Fig. 5:**
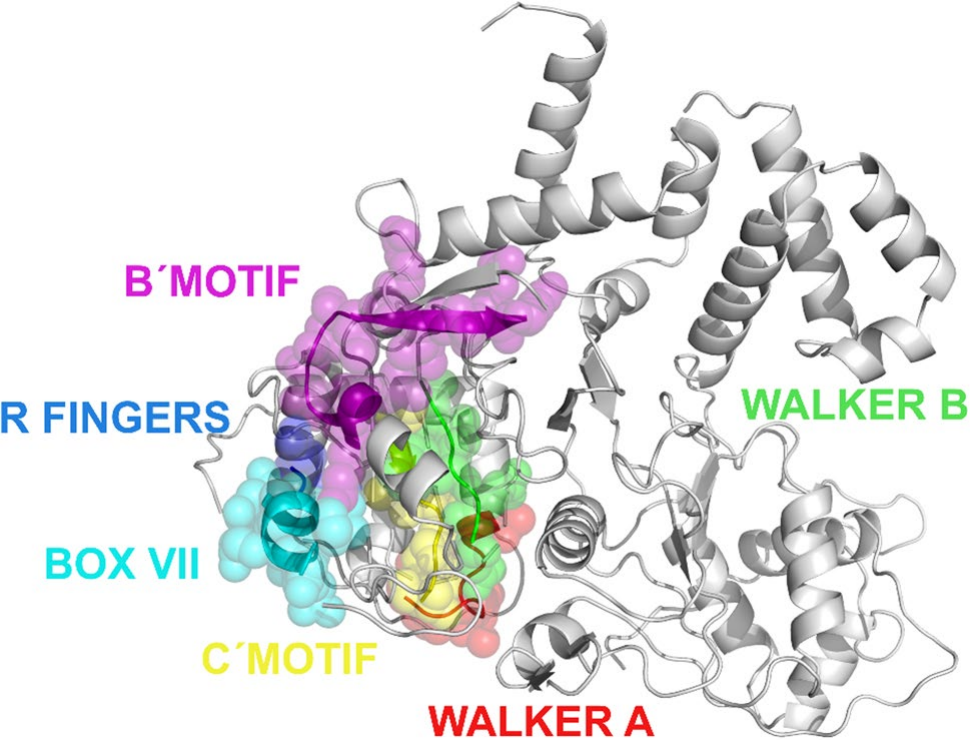
3-D modeling of an NS1 subunit of the Mexican PPV5 isolates. The conserved motifs Walker A, Walker B, B´ motif, C-motif, box VII, and the arginine fingers are shown. (PyMOL Molecular Graphics System, Version 2.0 Schrödinger, LLC)

**Fig. 6:**
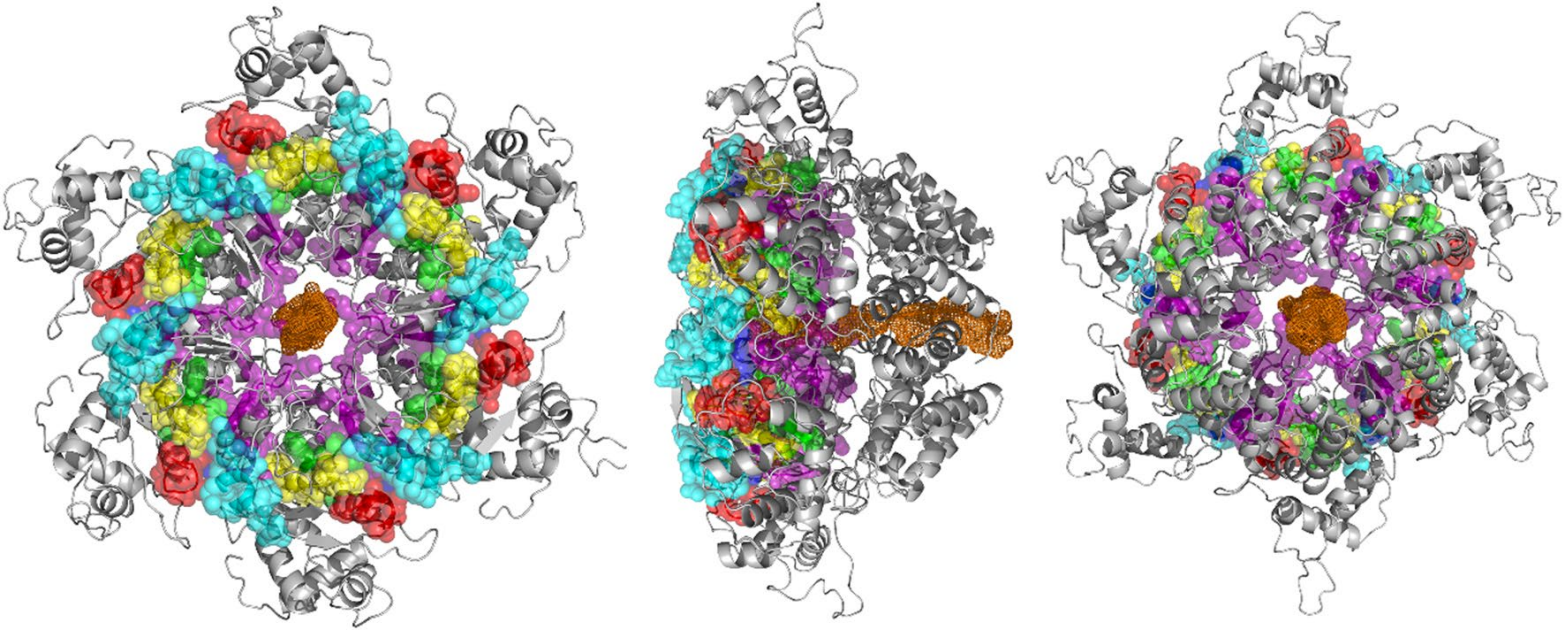
3-D modeling of the hexameric form of the NS1 protein of PPV5. The Walker A (red), Walker B (green), B´motif (purple), C-motif (yellow), box VII (light blue), and the arginine fingers (dark blue) are colored. Single-stranded DNA is shown in brown. Front (A), side (B), and back (C) views are presented. (PyMOL Molecular Graphics System, Version 2.0 Schrödinger, LLC)

A molecular model of an NS1 subunit of PPV5 showing the five conserved motifs of SF3 helicases is shown in Figure 5. A model of the functional hexameric complex, shown in Figure 6, shows the conserved motifs and the central core that incorporates the single-stranded DNA.

## Discussion

The PPV5 ORF1 is 1805 nt in length, encoding an NS1 protein of 601 aa that is highly conserved. NS1 plays a crucial role in viral DNA replication and transcription activation and also has cytotoxic effects [27]. This protein is used for taxonomic classification because it possesses highly conserved enzymatic domains (i.e., endonuclease and helicase). These conserved regions enable the alignment of amino acid sequences for reliable phylogenetic analysis [28, 29], and ≥ 80% of the ORF1 sequence is required for taxonomic classification [15]. Parvoviruses are considered members of the same species if their NS1 sequences are ≥ 85% identical, and members of the same genus if their NS1 proteins are at least 35–40% identical. If these criteria for categorization are not fulfilled, the genome length, transcription strategy, and the presence of additional protein coding genes are considered [2, 15].

In this study, 96.9% of ORF1 was sequenced, which exceeds the required threshold for phylogenetic studies. First, the PPV5 and PPV4 clades were found to group together in the same branch of the phylogenetic tree based on predicted NS1 amino acid sequences, with a bootstrap value of 100. A similar pattern was reported in two previous studies, with bootstrap values of 100 [30] and 96 [31], respectively, with this branch adjacent to PPV6 and porcine bocavirus 1 within the *Copiparvovirus* clade. This supports the notion of a close relationship between PPV5 and PPV4 [7].

Additionally, the Mexican PPV5 sequence OQ792211, which was obtained from a gilt blood sample collected in 2011, grouped together with two Chinese sequences (MZ577038 and MW853953) from diseased animals in 2016 [32] and 2017 [33]. This may be linked to the extensive commercial relationships between Mexico and China, resulting in the transfer of viruses between these countries, as suggested previously in retrospective studies on PPVs [12] and porcine anellovirus [34]. Interestingly, the identical Mexican sequences (OQ792212 and OQ792213) that clustered together in a separate branch originated from a PMWS outbreak in 2005, which was included in a retrospective study that found a potential relationship of PMWS to PPV5 [12]. Moreover, these differ in their amino acid sequences from all other PPV5 sequences, including the Mexican OQ792211 (Fig. 2, blue box), suggesting that they represent an endemic strain.

The Mexican PPV5 nucleotide sequences exhibited a high degree of similarity to each other and to PPV5 sequences from different countries (96.7% to 99.8%). Previous studies found 99.2% to 99.7% identity in ORF1 among PPV5 strains [5]. The Mexican PPV5 nucleotide sequences showed the closest similarity to PPV4 sequences. Previously, it was reported by Xiao et al. that the ORF1 sequences of these two viruses were 77.4% to 78.2% identical [7].

Likewise, the aa sequence identity of PPV5 compared to other PPVs was highest for PPV4 (86.2% to 87.3%), similar to the 84.6% to 85.1% range described by Xiao et al. [7], followed by PPV6 (54.9% to 55.4%) and BPV2 (38.6% to 38.8%). It is noteworthy that PPV4, PPV6, and BPV2 are members of the genus *Copiparvovirus* [2, 35] and that PPV5 was previously regarded as a copiparvovirus due to its strong relationship to PPV4 and PPV6 [7, 33, 36].

The results of the current study demonstrated that the Mexican PPV5 isolates from this study should be classified as members of the genus *Copiparvovirus* according to ICTV criteria. Despite the unique genome organization of PPV4, which includes an additional ORF designated as ORF3, similar to the bocaviruses, the genomes of PPV6 and BPV2, like PPV5, have two ORFs, reinforcing this proposal. Overall, the data presented herein agree with a previous study suggesting that PPV4, PPV5, PPV6, and BPV2 share a common ancestor and have a close evolutionary relationship [7]. Further comprehensive phylogenetic studies are still needed ascertain the taxonomy of PPV5.

Helicases are ATP-dependent enzymes that are classified based on their quaternary structure and movement direction [37]. Of the six helicase superfamilies, SF1 and SF2 are the most common [38, 39]. SF3 helicases have been identified in small ssDNA viruses, including coconut foliar decay virus (family *Geminiviridae*), adeno-associated virus (family *Parvoviridae*), minute virus of mice, and Southampton virus (family *Caliciviridae*) [37]. The SF3 Walker A motif sequence (GxxGxGKT) creates the ATP-binding loop through a conserved lysine [40, 41].

As mentioned above, the NS1 of *Parvoviridae* members has been identified as an SF3 helicase, but there have been a limited number of studies on its molecular characteristics. Most studies of NS1 have focused on pathogenic parvoviruses such as PPV1, concentrating on diagnosis and phylogeny [5–7, 11]. Additionally, the NS1 of CPV contains point mutations [14, 42], and such mutations in the ATP-binding site of the helicase domain have been shown to affect viral replication [22] However, those studies did not explore differences in conserved helicase motifs of emerging parvoviruses or analyze the three-dimensional structure of NS1.

In the current study, the Mexican NS1 sequences of PPV5 were found to contain the C-motif, which is characteristic of SF3 helicases, supporting its classification within this superfamily. Furthermore, although comparative analysis of PPV5, AAV, PPV1, and CPV sequences revealed a high level of nucleotide sequence similarity, some point mutations (missense mutations) [43] were identified in the regions encoding functional motifs of NS1. Like RNA viruses, which have high mutation rates due to a lack of a proofreading function in the RNA polymerase [44, 45], parvoviruses also have high mutation rates as well as the propensity to undergo recombination. It has been shown that a relatively small number of amino acid changes are needed to change the tissue tropism or host specificity of the virus [13, 46, 47].

Non-pathogenic viruses such as AAV and PPV5 have a threonine (T) at positions 4 and 8 in the Walker A motif, while pathogenic viruses such as PPV1 and CPV have a serine (S) at those positions. However, these differences are unlikely to affect the phosphorylation of this motif, since both T and S have been reported to be allowed at these positions [48]. Moreover, serine is also found in vaccine or attenuated strains of PPV1 and CPV, as shown in Figure 4. Similarly, the canonical SF3 C-motif of pathogenic PPV1 and CPV has an I→M substitution at position 3 (both apolar, aliphatic, and hydrophobic) and an S→T substitution at position 5, which are also similar (polar uncharged). Since both of these mutations are conservative substitutions, they are unlikely to affect the functionality or stability of the protein [49].

The conserved Walker B motif, which is composed of six amino acids, contains the residues EE in the active site at positions 5 and 6 in all sequences. These acidic residues, in conjunction with a cation such as Mg^+^, bind ATP. Notably, a tryptophan, which is apolar, aromatic, and hydrophobic, was observed at position 4 in the non-pathogenic viruses (PPV5 and AAV), while in the pathogenic viruses (PPV1 and CPV), there is isoleucine (apolar, aliphatic, and hydrophobic) at this position. Its proximity to the active site suggests that it could affect the enzyme's function [50]. Aromatic amino acids are predisposed to racemization, requiring less energy to undergo this process than aliphatic amino acids do. If racemization occurs in an active site of a molecule, it may have negative effects, such as reducing or nullifying its biological activity [51]. Thus, variations around the active site of the Walker B motif may alter the helicase activity of non-pathogenic viruses.

Notably, the E (negatively charged) → R (positively charged) change found in PPV5 may affect NS1 helicase function, as mutations in positively charged regions (R508 and R510) of CPV NS1 have been shown to prevent virion production [22]. This positively charged region also contains the arginine finger (position 9, Fig. 4), which is crucial for helicase function as a rotary motor, coordinating sequential hydrolysis through each subunit and assisting in oligomerization to create the characteristic hexameric ring of SF3 helicases [37, 40, 52].

In conclusion, missense mutations in pathogenic viruses may provide an advantage for viral adaptation [53], and mutations near or within the active site of NS1 can affect its function [50].

To our knowledge, no three-dimensional models of PPV5 NS1 have been published previously. Our 3D model showed all of the conserved motifs of SF3 helicases, and a model of the functional hexamer was also constructed.

Because some antiviral medications for use in humans inhibit helicase activity [54, 55], the characterization of helicases from viruses infecting animals may be useful for designing antiviral drugs for both human and veterinary medicine. The present work provides insights regarding the possible implications of missense mutations in the NS1 helicase of PPV5.

## Data Availability

The datasets generated during and/or analyzed in the current study are available in the National Center of Biotechnology Information [NCBI] repository (https://www.ncbi.nlm.nih.gov/) under the accession numbers OQ792211, OQ792212, and OQ792213.
